# Quantification and Genotyping of Norovirus in Aerosols from Wastewater Treatment Plants in Thailand

**DOI:** 10.1007/s12560-025-09647-1

**Published:** 2025-05-23

**Authors:** Leera Kittigul, Kitwadee Rupprom, Yuwanda Thongpanich, Thanakrit Neamhom, Fuangfa Utrarachkij

**Affiliations:** 1 Department of Microbiology, Faculty of Public Health, Mahidol University, 420/1 Ratchawithi Road, Bangkok, 10400 Thailand; 2 Department of Clinical Pathology, Faculty of Medicine Vajira Hospital, Navamindradhiraj University, Bangkok, Thailand; 3 Department of Environmental Health Sciences, Faculty of Public Health, Mahidol University, Bangkok, Thailand

**Keywords:** Norovirus, Rotavirus, Aerosol, Wastewater treatment plant, Quantification, Genotype

## Abstract

Norovirus and rotavirus are the major causes of acute gastroenteritis in humans worldwide. Due to their small size, these enteric viruses present in wastewater become aerosolized. The objective of this study was to assess the presence and concentrations of norovirus and rotavirus in aerosol samples collected from wastewater treatment plants (WWTPs) in Thailand. A developed method for concentrating viruses and performing molecular detection was used to determine naturally occurring enteric viruses. Of the 24 collected aerosol samples, 8 (33.3%) and 2 (8.3%) tested positive for norovirus RNA using RT-qPCR and RT-nested PCR, respectively. Based on RT-qPCR, norovirus GII RNA was detected more frequently in aerosol samples (7/24, 29.2%) compared to norovirus GI RNA (2/24, 8.3%). The norovirus GI concentrations were 9.8 × 10^2^ and 3.2 × 10^3^ genome copies/m^3^. The norovirus GII concentrations ranged from 1.5 × 10^2^ to 5.5 × 10^3^ genome copies/m^3^. RT-nested PCR detected norovirus GII RNA and the rare GII.21 norovirus strains were identified in the two aerosol samples. However, rotavirus RNA was not detected in any of the aerosol samples using either RT-qPCR or RT-nested PCR. This study highlights the quantification and genotyping of norovirus in aerosol samples generated from wastewater, suggesting a potential effect of airborne transmission for WWTPs workers.

**Supplementary Information:**

The online version contains supplementary material available at 10.1007/s12560-025-09647-1.

## Introduction

Human norovirus is the leading cause of epidemic and sporadic gastroenteritis in both children and adults worldwide. The illness can be severe in young children, the elderly, and immunocompromised individuals (Shah & Hall, 2018). Noroviruses are RNA viruses belonging to the family *Caliciviridae*. They are currently subdivided into ten genogroups (GI−GX), comprising at least 49 genotypes and 60 P-types. Genogroup II genotype 4 (GII.4) is a predominant genotype that causes acute gastrointestinal illness in humans (Chhabra et al., 2019). Norovirus is transmitted via the fecal-oral route through contact with infected persons, contaminated surfaces or ingestion of contaminated food or water (de Graaf et al., 2016). Norovirus spreads most easily due to high viral loads in stools (10^5^−10^9^ copies/g) and vomit (10^3^−10^6^ copies/g), and high stability in the environment (La Rosa et al., 2013; Teunis et al., 2015). Due to their small size, norovirus infectious particles presumably become aerosolized from vomit (Kirby et al., 2016) or toilet flushing (Boles et al., 2021; Johnson et al., 2013), settling in the upper respiratory tract during inhalation, and swallowed. Airborne transmission of norovirus has been suggested, and has been suspected in outbreaks in healthcare facilities (Bonifait et al., 2015), hospitals (Alsved et al., 2020), and a kindergarten (Zhang et al., 2017). Additionally, norovirus RNA was demonstrated in air samples collected from wastewater treatment plants (WWTPs) (Masclaux et al., 2014; Matsubara & Katayama, 2019; Stobnicka-Kupiec et al., 2022).

Rotavirus is the primary cause of acute diarrhea-related morbidity and mortality in infants and young children under five years of age worldwide (Omatola & Olaniran, 2022). According to rotavirus surveillance data in Southeast Asia from 2008 to 2018, 40.8% of all diarrheal illnesses in children were due to rotavirus infection (Lestari et al., 2020). In Thailand, community surveillance found rotavirus infection rates ranging from 13.2 to 24.2% among children under five years old (Lestari et al., 2020), while hospital-based surveillance reported higher rates of 28.4 to 44.5% (Maneekarn & Khamrin, 2014). Among adult patients with acute gastroenteritis admitted to hospitals, a lower detection rate (7.4%) of rotavirus infection was observed (Satayarak et al., 2020). Rotaviruses are non-enveloped RNA viruses with a diameter of 70 nm that belong to the *Reoviridae* family and genus *Rotavirus*. Their genome consists of 11 double-stranded RNA segments that are enclosed in a triple-layered capsid. There are currently at least 36 G genotypes and 51 P genotypes known in both humans and animals (Sadiq et al., 2018). Transmission of rotavirus occurs primarily through the fecal-oral route, directly from person-to-person contact or indirectly by contaminated fomites. While less often investigated, the presence of rotavirus has been detected in air samples collected from WWTPs (Brisebois et al., 2018; Stobnicka-Kupiec et al., 2022).

WWTPs involve various mechanical motions that generate bubbles and fine droplets, potentially aerosolizing viruses present in wastewater by bubble bursting, mechanical motion, and water turbulence. Aeration systems, treatment sections, and natural conditions may affect the presence of bioaerosols. Inhalation or ingestion following direct contact of these aerosols may be a significant contributor to the occurrence of bioaerosol-induced gastrointestinal diseases (Lou et al., 2021). Gastrointestinal symptoms caused by viruses in WWTP workers have been partly attributed to the work-related illness (Masclaux et al., 2014). Therefore, assessing the occupational exposure risks of viral gastroenteritis among WWTP workers is an important public health concern.

Our previous study in Thailand revealed a high prevalence of enteric viruses, such as norovirus and rotavirus in recycled water and sewage sludge samples derived from WWTPs (Kittigul & Pombubpa, 2021; Kittigul et al., 2019). It is of interest to monitor the presence of these enteric viruses in aerosols generated from WWTPs. The objective of this study was to determine the potential airborne transmission of gastrointestinal viruses by determining the presence of norovirus and rotavirus in air samples collected at WWTPs. We utilized a developed method for virus concentration and molecular quantification of virus-laden aerosols.

## Materials and Methods

### Air Sampling

**Fig. 1:**
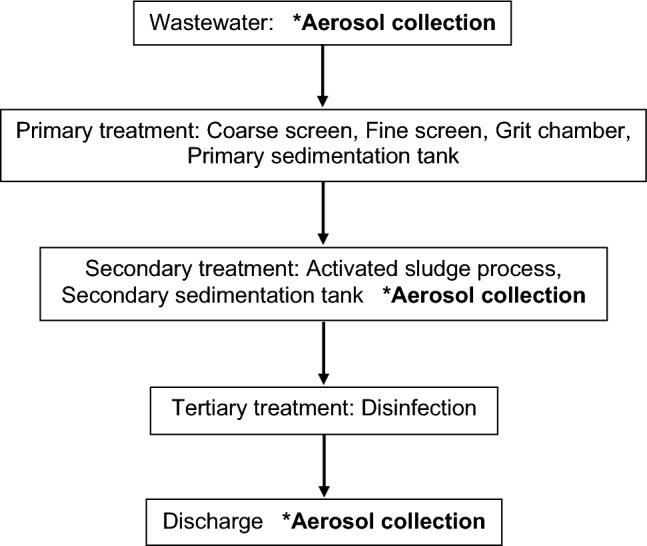
Wastewater treatment processes and sites of aerosol collection in a wastewater treatment plant

A total of 24 aerosol samples were collected from eight WWTPs of the Department of Drainage and Sewerage, Bangkok, Thailand from September to October 2023. All of these WWTPs are managed by the Department of Drainage and Sewerage, government-run facilities responsible for the drainage, flood protection and wastewater disposal. The WWTPs used a series of physical, chemical and biological process to remove solid waste, particles and organic matter, and to reduce microorganisms. These processes included preliminary, primary, secondary and tertiary treatments. Wastewater treatment system utilized in each WWTP was contact stabilization activated sludge, two stage activated sludge, cyclic activated sludge systems (CASS), vertical loop reactor activated sludge (VLR-AS) or activated sludge with nutrients removal. The aerosol samples were collected above/near the wastewater stream from three sampling points at each WWTP per day: the wastewater entrance, the treatment site, and the exit site before being discharged into a canal. The diagram of treatment processes and sampling sites of aerosol collection is shown in Fig. 1.

Aerosol samples were collected using the BioSampler (SKC Inc., Eighty-Four, PA, USA) with a BioLite air sampling pump according to a previous study which evaluated the efficiency for norovirus collection in aerosols (Rupprom et al., 2024a). The impinger biosampler was calibrated for a flow rate of 12.5 L/min, 30 min at a height of 1 m above ground level. The total air volume collected was 375 L or 0.375 m^3^, with aerosol samples collected in 5 mL of sterile distilled water via collection vessels. Over a two-month period, three air samples were taken from each WWTP once a week and transported to a laboratory for immediate processing for virus concentration within the same day of sample collection.

### Virus Concentration

Aerosol sampling in sterile distilled water was processed by speedVac centrifugation using a vacuum centrifuge (UNIEQUIP Laborgeratebau und-vertriebs GmbH, Munich, Germany) to reduce the volume to 600–650 μL for 2 h (Rupprom et al., 2024a). The concentrated samples were stored at − 80 °C until used for RNA extraction.

### RNA Extraction and RT-qPCR

All concentrated aerosol samples (200 μL) underwent RNA extraction using QIAamp Viral RNA Mini Kit (Qiagen, Hilden, Germany) in accordance with the manufacturer’s protocol, resulting in a final elution volume of 60 μL. RT‐qPCR was then performed to quantify the extracted viral RNA for norovirus GI and norovirus GII in separate strips. The one-step RT-qPCR was carried out in 20 μL reaction mixture using the LightCycler RNA Master Hybprobe with *Tth* DNA polymerase (Roche Diagnostics, Mannheim, Germany), in accordance with the manufacturer’s instructions with minor modifications. The one-step RT-qPCR conditions were optimized for detecting norovirus GI as previously described in the literature (Rupprom et al., 2018). Briefly, a 5 μL of the RNA extract was mixed with 15 μL of the RT-qPCR reaction mixture containing 1X LightCycler RNA Master Hybprobe, *Tth* DNA polymerase reaction buffer, dNTP mix (with dUTP instead of dTTP), 3.25 mM Mn (OAc)_2_, 0.4 µM each of primers GITF and GITR, 0.2 µM of probe GIT-TP (Table S1) and nuclease-free water. The thermal cycling conditions were as follows: reverse transcription at 58 °C for 30 min; initial denaturation at 95 °C for 4 min; 45 cycles of denaturation at 95 °C for 15 s and annealing/extension at 55 °C for 1 min on the LightCycler 96 Real-Time PCR instrument (Roche Diagnostics).

For norovirus GII, a 5 μL of the RNA extract was mixed with 15 μL of the RT-qPCR reaction mixture, which included 1X LightCycler RNA Master Hybprobe, *Tth* DNA polymerase reaction buffer, dNTP mix (with dUTP instead of dTTP), 3.25 mM Mn (OAc)_2_, 0.5 µM of primer QNIF2, 0.9 µM of primer COG2R, 0.25 µM of probe QNIFs (ISO, 2017) (Table S1) and nuclease-free water. The one-step RT-qPCR conditions were optimized previously for the detection of norovirus GII (Rupprom et al., 2018). The RT-qPCR thermal cycling conditions were as follows: 55 °C for 30 min; 95 °C for 5 min; 45 cycles of 95 °C for 15 s and 60 °C for 1 min on the LightCycler 96 Real-Time PCR instrument.

RNA transcripts of norovirus GI and GII were prepared using in vitro RNA transcription (Rupprom et al., 2018). Briefly, norovirus RNA targets were amplified by RT-PCR using primers G1 FF1, G1 FF2, G1 FF3, and G1-SKR for GI and G2 FB1, G2 FB2, G2 FB3, and G2-SKR for GII. Amplicons (597 bp of GI; 468 bp of GII) were inserted into pCR™4 TOPO® vector (Invitrogen, Carlsbad, CA) and transfected into One-shot TOP10 *Escherichia coli* (Invitrogen). Recombinant plasmids were linearized with *Mss*I. Then, in vitro RNA transcription was performed using RiboMAX™ Large Scale RNA Production Systems-T7 (Promega, Madison, WI). A standard curve was generated from a tenfold dilution series of norovirus GI or GII RNA transcripts, ranging from 1 × 10^3^–1 × 10^7^ RNA copies/mL. Using RT-qPCR, quantification cycle (Cq) values were plotted against log norovirus GI or GII RNA concentrations. Quantification of norovirus genome copy numbers in the aerosol samples was achieved by comparing the obtained Cq values with the constructed standard curve. The concentrations of norovirus GI and GII in aerosol samples were quantified by RT-qPCR as genome copies/mL. The genome copies/mL were converted to be genome copies/m^3^ of air (Rupprom et al., 2024a).

### RT-nested PCR for Detection of Norovirus

The RNA extract was tested for the presence of norovirus GI and GII using RT-nested PCR as described by Tunyakittaveeward et al. (2019). For each virus genogroup, a 2 μL of the RNA extract was added to the RT-PCR mixture (48 μL) using the SuperScript III One-Step RT-PCR System with Platinum *Taq* DNA polymerase (Invitrogen, Carlsbad, CA) in separate tubes. Primer GI (COG1F and G1-SKR) or GII (COG2F and G2-SKR) (Table S1) was used for RT-PCR. After RT-PCR amplification, primer GI (G1-SKF and G1-SKR) or GII (G2-SKF and G2-SKR) (Table S1) was used for nested PCR. The amplification reaction was performed on the BIO-RAD T100 Thermal cycler (Applied Biosystems, Foster City, CA, USA) using established thermocycling profiles. In the RT-PCR for norovirus GI and GII, the cycling conditions consisted of RT at 42 °C for 60 min; 94 °C for 2 min; PCR of 35 cycles at 94 °C for 1 min, 50 °C for 1 min, 72 °C for 1 min; and final extension at 72 °C for 3 min. In the nested PCR, the cycling conditions were set at 94 °C for 3 min; PCR of 35 cycles for norovirus GI or PCR of 30 cycles for norovirus GII at 94 °C for 1 min, 50 °C for 1 min, 72 °C for 2 min; and final extension at 72 °C for 15 min. By agarose gel electrophoresis, the amplicon sizes of norovirus GI and GII were found to be 330 bp and 344 bp, respectively.

### RT-qPCR for Rotavirus

Rotavirus NSP3 primers and probe were used to target at the highly conserved region of the NSP3 gene of rotavirus. The one-step RT-qPCR conditions using these primers and probe were described by Zeng et al. (2008). A modified RT-qPCR was performed in 20 µL containing 15 µL of the reaction mixture and 5 µL of the extracted RNA. The reaction mixture included 1X LightCycler RNA Master Hybprobe, *Tth* DNA polymerase reaction buffer, dNTP mix (with dUTP instead of dTTP), 3.25 mM Mn (OAc)_2_, 0.4 µM of the forward and reverse primers (NSP3F and NSP3R), 0.2 µM of the probe (NSP3P) (Table S2) and nuclease-free water. The reaction PCR tube was placed into the LightCycler 96 Real-Time PCR instrument. RT-PCR cycling conditions were as follows: reverse transcription at 48 °C for 30 min; initial denaturation at 95 °C for 10 min, followed by PCR (45 cycles) at 95 °C for 15 s, and annealing/extension at 60 °C for 1 min.

A standard curve was generated from a tenfold dilution series of rotavirus RNA transcripts, ranging from 10^3^ to 10^7^ genome copies/mL prepared by in vitro RNA transcription. Using RT-qPCR, Cq values were plotted against log rotavirus RNA concentrations. The rotavirus genome copy numbers in the aerosol samples were quantified by comparing obtained Cq values to the constructed standard curve.

### RT-nested PCR for Detection of Rotavirus

Group A rotavirus RNA was amplified by RT-nested PCR assay as described previously (Kittigul & Pombubpa, 2021) using VP7 specific primers: RV1 and RV2 for RT-PCR; and RV3 and RV4 for nested PCR (Table S2). In the first-round (RT-PCR), the cycling conditions consisted of RT at 41 °C for 60 min; 94 °C for 2 min, PCR of 25 cycles at 94 °C for 30 s, 55 °C for 30 s, and 72 °C for 60 s, concluding with a final extension at 72 °C for 3 min. In the second-round (nested PCR), the RT-PCR product was further amplified under the same conditions as the first-round PCR excluding the reverse transcription step, but for 40 cycles. An amplicon size of 346 bp was considered indicative of rotavirus presence.

### RT-PCR Inhibition Test

To test RT-PCR inhibition in concentrated water samples prepared from aerosol samples and subjected to RNA extraction, norovirus GI, norovirus GII or rotavirus RNA transcripts with known concentrations were used as external controls. A volume of 1 μL aliquot of external control (EC RNA) at a concentration of 10^4^ RNA copies/μL was added to either 5 μL of the water concentrate RNA, refered to as sample RNA, or 5 μL of PCR grade water, refered to as water RNA. A volume 14 μL of reaction mixture was then added to the sample RNA or water RNA, totaling a final volume of 20 μL. RT-qPCR was performed for norovirus GI, norovirus GII or rotavirus as previously described. The RT-PCR inhibition was calculated according to the following equation:

$${\text{RT}}\, - \,{\text{PCR}}\,{\text{ inhibition}}\, = \, \, (1 - 10^{{\Delta{\text{Cq}}/{\text{m}}}} )\, \times \,100\%$$

where ΔCq = Cq value (sample RNA + EC RNA) − Cq value (water RNA + EC RNA) and m = slope of the standard curve. The acceptable level of RT-PCR inhibition was ≤ 75% according to the value recommended in the ISO standard procedure for quantitative detection of norovirus in food and water (ISO, 2017).

### Phylogenetic Analysis

The nested PCR amplicons of the norovirus GII-positive aerosol samples underwent DNA sequencing, and the resulting nucleotide sequences were aligned with reference norovirus strains available in the GenBank database using the BLAST server. Phylogenetic analysis of the norovirus was conducted using MEGA 11.0 software. The study strain sequences and reference sequences retrieved from GenBank were aligned in MEGA and the phylogenetic tree was constructed using the neighbour-joining method based on the Tamura-Nei model with bootstrap analysis performed over 1000 replicates. Two nucleotide sequences of norovirus GII, coded AIR-WW09 and AIR-WW11, were submitted to the GenBank database and assigned the accession numbers PP976430 and PP976431, respectively.

## Results

### Detection of Noroviruses in the Aerosol Samples

A total of 24 aerosol samples were collected from 8 WWTPs and tested for norovirus RNA and rotavirus RNA (Table S3). Undiluted RNA extract, 1:4 and 1:10 dilutions were tested by RT-qPCR and the dilution at 1:4 provided the highest frequency of norovirus detection. Norovirus RNA was detected in 8/24 samples (33.3%) using RT-qPCR and in 2/24 samples (8.3%) using RT-nested PCR. Based on RT-qPCR, norovirus GII RNA was detected more frequently (7/24, 29.2%) than norovirus GI RNA (2/24, 8.3%). One aerosol sample collected at the entrance site was found positive for both norovirus GI and GII. Norovirus RNA was detected at the treatment site with the highest frequency (5/8, 62.5%), followed by the exit site (2/8, 25%) and the entrance site (1/8, 12.5%). Using RT-nested PCR, norovirus GII RNA was detected in the two aerosol samples which were also positive by RT-qPCR and collected at the treatment and exit sites of WWTPs. Rotavirus RNA was not detected in any aerosol samples using either RT-qPCR at undiluted, 1:4 and 1:10 dilution of RNA extract or RT-nested PCR (Table 1 and Table S4).

**Table 1:** Test of enteric virus RNA in aerosol samples collected from WWTPs using two molecular methods

Molecular method	No of samples	Norovirus, No. (%)				Rotavirus
		GI only	GII only	GI + GII	Total	No. (%)
RT-qPCR	24	1 (4.2)	6 (25.0)	1 (4.2)	8 (33.3)	0 (0)
RT-nested PCR	24	0 (0)	2 (8.3)	0 (0)	2 (8.3)	0 (0)

### Quantification of Noroviruses in the Aerosol Samples

Of the 8 norovirus-positive aerosol samples, viral loads could be determined for 2 samples of GI and 5 samples of GII. The norovirus GI concentrations were measured at 1.9 × 10^3^ and 6.2 × 10^3^ genome copies/mL, which correspond to 9.8 × 10^2^ and 3.2 × 10^3^ genome copies/m^3^, respectively. For norovirus GII, the concentrations ranged from 3.2 × 10^2^ to 1.1 × 10^4^ genome copies/mL, equivalent to 1.5 × 10^2^–5.5 × 10^3^ genome copies/m^3^. Additionally, weakly positive GII fluorescence signals were detected in two aerosol samples, in which the viral load could not be determined and both exhibited RT-PCR inhibition exceeding the acceptable level of ≤ 75%. Nevertheless, three aerosol samples with RT-PCR inhibition higher than the acceptable level tested positive for norovirus GII and could be consequently quantified for genome copies. All 8 aerosol samples examined for norovirus GI had RT-PCR inhibition within the acceptable level. Two aerosol samples, each collected on the same day from different sampling points in each WWTP (AW08 and AW09; AW11 and AW12), consistently tested positive for norovirus GII, one quantifiable and the other yielding a weak signal, and RT-PCR inhibition results higher than the acceptable level (Table 2).

**Table 2:** Norovirus-positive aerosol samples from WWTPs detected and quantified using RT-qPCR

No	Sample code	Collection date	Norovirus GI			Norovirus GII		
			GC^a^/mL	GC/m^3^	RT-PCR inhibition (%)	GC^a^/mL	GC/m^3^	RT-PCR inhibition (%)
1	AW01	13 Sep 23	1.9 × 10^3^	9.8 × 10^2^	6.0	1.1 × 10^4^	5.5 × 10^3^	30.0
2	AW05	25 Sep 23	–	–	15.8	4.6 × 10^3^	2.2 × 10^3^	78.4
3	AW08	27 Sep 23^b^	–	–	22.8	2.5 × 10^3^	1.3 × 10^3^	83.5
4	AW09^d^	27 Sep 23^b^	–	–	37.0	±	±	80.3
5	AW11^d^	29 Sep 23^c^	–	–	20.4	3.2 × 10^2^	1.5 × 10^2^	93.9
6	AW12	29 Sep 23^c^	–	–	16.4	±	±	84.2
7	AW14	04 Oct 23	–	–	25.6	2.3 × 10^3^	1.2 × 10^3^	54.8
8	AW17	06 Oct 23	6.2 × 10^3^	3.2 × 10^3^	36.6	–	–	20.2

^a^GC/mL refers to the concentrations of viral genome copies/mL in RNA extract tested using RT-qPCR and multiplied by a dilution factor (1:4 dilution of RNA extract)

^b,c^The aerosol samples were collected from the same WWTP: AW08 and AW09; AW11 and AW12

^d^The aerosol samples were also positive for norovirus GII using RT-nested PCR

A negative result is indicated by “–”. A weak fluorescence signal (0.01–0.05) result is indicated by “ ± ”. The acceptable level of RT-PCR inhibition is set at ≤ 75%

### Molecular Characterization of Norovirus GII

**Fig. 2:**
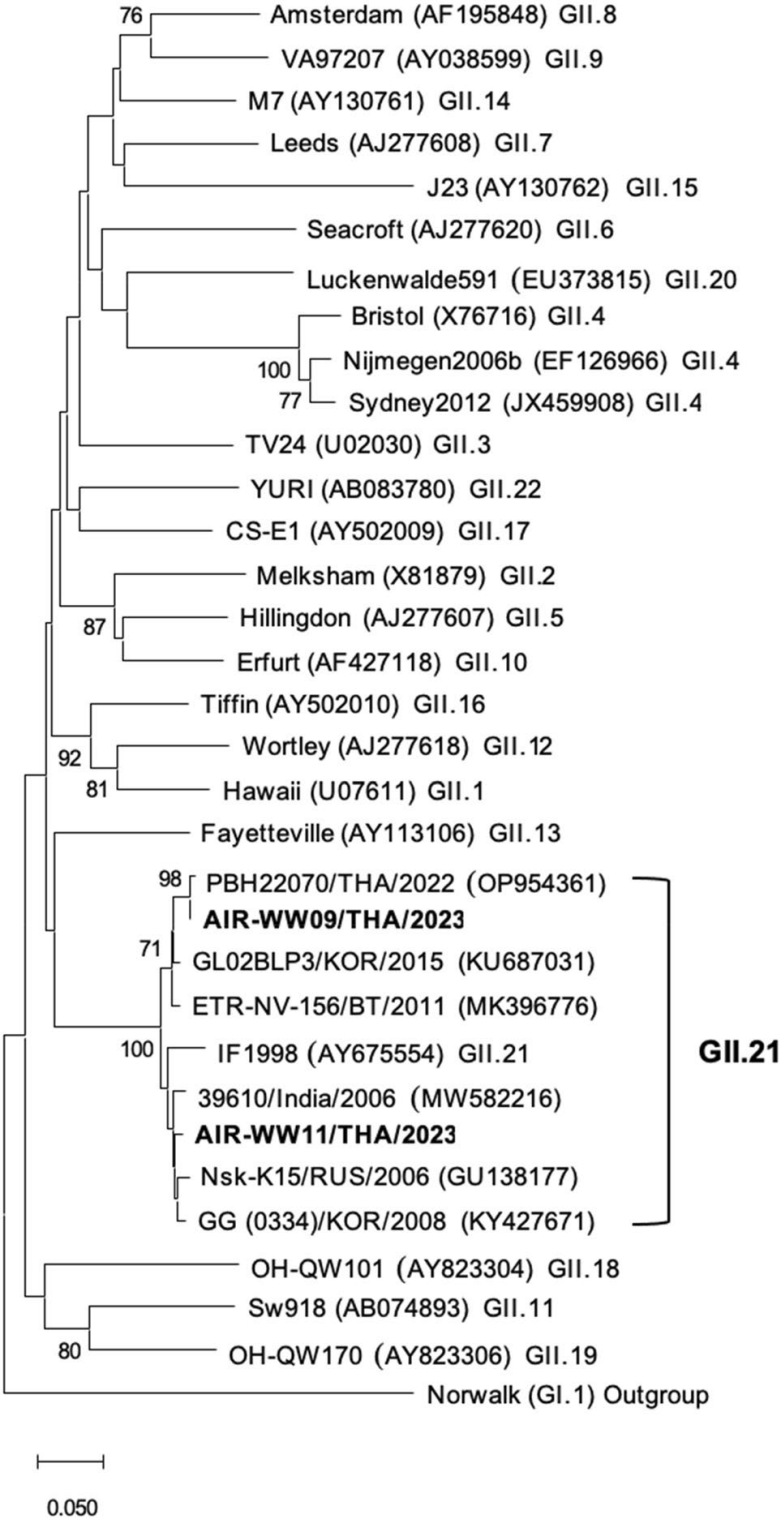
Phylogenetic analysis of the norovirus GII strains detected by RT-nested PCR. The partial nucleotide sequence (291 bp) was compared to the reference strains. The phylogenetic tree of norovirus GII strains detected in this study (AIR-WW09/THA/2023 and AIR-WW11/THA/2023) and the reference sequences retrieved from GenBank was constructed using the neighbour-joining method based on the Tamura-Nei model with bootstrap analysis of 1,000 replicates. Numbers at each node (> 70) are shown. The nucleotide sequences obtained in this study are indicated in bold. The scale bars indicate the nucleotide distance

The two norovirus GII-positive aerosol samples detected by RT-nested PCR were subjected to DNA sequencing of the amplicons. Nucleotide sequence alignment of the aerosol samples using BLAST program revealed that the sequences from samples AIR-WW09 (coded AW09) and AIR-WW11 (coded AW11) exhibited 99.7% and 98.8% nucleotide identity with PBH22070/THA/2022 (OP954361) and GG (0334)/KOR/2008 (KY427671), respectively. Phylogenetic analysis confirmed that both sequences clustered in the reference GII.21 strain (IF1998, AY675554), classifying them as belonging to the GII.21 norovirus genotype. These strains were closely related to the GII.21 strains previously detected in stool samples from patients with acute gastroenteritis in Thailand, Korea, Bhutan, India, and Russia (Fig. 2). The two GII.21 samples; AIR-WW09 and AIR-WW11 were collected from different WWTPs on 27 Sep 23 and 29 Sep 23. Both samples tested positive for norovirus GII using RT-qPCR. Sample AIR-WW09 exhibited relatively low fluorescence signal, whereas the sample AIR-WW11 contained norovirus GII at a concentration of 1.5 × 10^2^ genome copies/m^3^ (Table 2).

### RT-PCR Inhibition in the Air Samples

The use of undiluted RNA extract from the water concentrates of aerosol samples revealed RT-PCR inhibition was higher than the acceptable level (≤ 75%) for both norovirus and rotavirus. However, at a 1:4 dilution of RNA extract, RT-PCR inhibition fell within the acceptable levels for norovirus GI (100% of samples), with higher frequency than norovirus GII (66.7% of samples) and rotavirus (25% of samples). This suggests that the water concentrates from aerosol samples showed the highest degree of RT-PCR inhibition for rotavirus, followed by norovirus GII and then norovirus GI (Table 3). Further dilution to 1:10 of RNA extract resulted in acceptable RT-PCR inhibition levels for all aerosol samples tested for spiking norovirus GII and rotavirus transcripts but provided negative results for these enteric viruses in sample tests.

**Table 3:** RT-PCR inhibition of enteric viruses in aerosols samples from WWTPs using RT-qPCR

Enteric virus	RT-PCR inhibition; RNA extract at 1:4 dilution			
	Acceptable (≤ 75%)		Unacceptable (> 75%)	
	No./Total (%)	Mean ± SD	No./Total (%)	Mean ± SD
Norovirus GI	24/24 (100)	21.0 ± 22.2	0 (0)	–
Norovirus GII	16/24 (66.7)	34.8 ± 20.3	8/24 (33.3)	83.2 ± 5.6
Rotavirus	6/24 (25.0)	68.9 ± 4.6	18/24 (75.0)	85.1 ± 7.0

## Discussion

Bioaerosols generated from wastewater in WWTPs can be a potential source of viral gastrointestinal diseases leading to occupational risk for plant workers. Building on a previously developed virus concentrating and detection method for norovirus GII in a controlled aerosol chamber in the laboratory, air sampling collection in distilled water of 5 mL using the SKC BioSampler in spiking experiments provided a higher recovery of norovirus GII RNA and could be performed in shorter collection time than 20 mL. Based on the evaluated virus concentrating method by speedVac centrifugation and molecular detection, the concentrations of norovirus GII RNA recovered from air samples in the aerosol chamber were in the range of 10^2^ to 10^5^ genome copies/mL (Rupprom et al., 2024a). Therefore, in the present study we collected aerosol samples in distilled water of 5 mL using the SKC BioSampler and processed for virus concentration. RT-qPCR and RT-nested PCR methods, which were established in our laboratory for environmental samples (Kittigul & Pombubpa, 2021; Kittigul et al., 2019), were also employed to quantify and identify for genotype of naturally occurring enteric viruses, including norovirus and rotavirus in aerosol samples collected from WWTPs.

In Thailand, there is no obvious seasonality of norovirus prevalence. The virus could be detected in patients with acute gastroenteritis all year round with peaks in winter and summer (Phengma et al., 2022). Additionally, norovirus can be found in environmental samples continually throughout the year (Kittigul et al., 2019). In this study, the aerosol samples were collected from September to October since the prevalence of norovirus tended to increase during late rainy season and early winter. We found a predominance of norovirus GII in the collected aerosol samples corresponding to a high prevalence of norovirus GII as reported in previous studies of patients with acute gastroenteritis (Chiu et al., 2022; Eftekhari et al., 2023) and wastewater samples (Valdivia-Carrera et al., 2023). The previous study of norovirus in different WWTPs in Bangkok Metropolitan Region, Thailand reported the GI predominance in recycled water and the GII predominance in sewage sludge (Kittigul et al., 2019). We found norovirus GI RNA present in the aerosol samples at a lower frequency than GII. It seems that the presence of predominant norovirus genogroup varied upon environmental resistance for types of collected samples i.e. GI predominance in recycled water, whereas GII predominance in sewage sludge and aerosol. The stability of human norovirus genogroup in environment needs to be further studied, however, the difficulty in cultivation of the virus in tissue culture system (Ettayebi et al., 2024) may be an obstacle of environmental resistance study. Regarding the sites of aerosol sample collection in WWTPs, the aerosol samples collected at the wastewater treatment sites exhibit a high frequency of norovirus detection probably due to mechanical motion, water turbulence, and aeration systems generating virus-laden aerosols.

Our findings of 10^2^ to 10^3^ genome copies/m^3^ of norovirus GI and norovirus GII RNAs in the aerosol samples align with a previous study that reported noroviruses with the concentrations up to 10^3^ genome copies/m^3^ in air samples collected from WWTPs (Stobnicka-Kupiec et al., 2022). However, a study in the cities with poor sanitation found a predominance of norovirus GI with higher average viral load of 320 genome copies/m^3^ than norovirus GII in aerosol samples (Ginn et al., 2021). This difference may be attributed to the differences in geographical areas, the wastewater treatment systems, and environmental factors such as the outdoor environment, as noted in the previous study (Ginn et al., 2021).

Although a longitudinal study in Thailand exhibited the prevalence of rotavirus in recycled water and sewage sludge samples derived from WWTPs (Kittigul & Pombubpa, 2021), rotavirus RNA was not found in any aerosol samples in this study using the same virus concentrating method by speedVac centrifugation and molecular detection by RT-nested PCR. The developed method for rotavirus detection was previously validated using environmental water samples (Kittigul et al., 2005). However, the validation of rotavirus in aerosol samples has not yet been studied. We could not detect rotavirus in the collected aerosol samples probably due to the extremely low viral load in aerosols or the presence of RT-PCR inhibitors (Schrader et al., 2012). It appears that RT-PCR inhibitors in water concentrates from air sampling may affect the amplification of rotavirus RNA more than norovirus GII, and norovirus GI. RNA extract at 1:4 dilution is suitable for virus detection using RT-qPCR consistent with our previous study on norovirus in aerosols from a hospital (Rupprom et al., 2024b). The high airflow rate of aerosol collection, the long sampling period in conjunction with the virus processing methods can potentially damage viral particles. RNAs may degrade and become unstable due to the RNase enzyme present in the environment (Brisebois et al., 2018). Previous studies reported rotavirus detection in the air samples collected from WWTPs (Brisebois et al., 2018; Pasalari et al., 2019; Stobnicka-Kupiec et al., 2022). The difference may be partly due to different places of aerosol sample collection, RT-PCR inhibitors, and methods for rotavirus detection.

The low viral load in aerosol samples make norovirus identification challenging. Only a few reports of norovirus genotypes in aerosols have been documented. Norovirus GII.4 Sydney strains were identified in the air samples from hospital outbreaks (Alsved et al., 2020). Our previous study successfully identified norovirus GII.17 strains in the aerosol samples collected from a tertiary care hospital in Thailand (Rupprom et al., 2024b). This is the first study to demonstrate the presence of rare norovirus GII.21-laden aerosols obtained from WWTPs. Occupational exposure to bioaerosols may affect the health of workers in WWTPs. The two GII.21 strains (AIR-WW09 and AIR-WW11) were detected from separate WWTPs with different sampling times, thereby indicating the distribution of norovirus GII.21 in aerosol samples. Notably, both AIR-WW09 and AIR-WW11 tested positive for norovirus GII using both RT-qPCR and RT-nested PCR methods. RT-qPCR was utilized for viral quantification while RT-nested PCR was employed for genotyping. This study demonstrates the superior performance of RT-qPCR over RT-nested PCR for norovirus detection. Further studies utilizing viability RT-qPCR, which can differentiate viable and non-viable norovirus, would be valuable for a more comprehensive interpretation of PCR results. Norovirus GII.21 RNAs were also reported in stool and/or water samples from Bhutan (Yahiro et al., 2015), Ethiopia (Tegegne et al., 2024), and Thailand (Kumthip et al., 2024; Udompat et al., 2024), confirming the distribution of this strain in humans and the environment.

The findings of norovirus in aerosol samples collected from WWTPs support the risk of airborne norovirus exposure for wastewater workers given the low infectious dose and environmental resistance (La Rosa et al., 2013; Teunis et al., 2008). Exposure to airborne norovirus may pose a susceptible health risk to workers inside the WWTP (Uhrbrand et al., 2017) especially for immunocompromised individuals. The current study demonstrates that the quantitative detection and genotyping of noroviruses at WWTP workplaces using molecular tools are crucial for exposure assessment and should be included in risk management strategies for occupational environments where wastewater-derived viruses are present.

## Supplementary Information

Below is the link to the electronic supplementary material.

Supplementary file1 (DOCX 35 KB)

## Data Availability

No datasets were generated or analysed during the current study.
