# Supplementary material for: Quantification and Genotyping of Norovirus in Aerosols from Wastewater Treatment Plants in Thailand
Source: Food Environ Virol. 2025 May 23;17(2):31. doi: 10.1007/s12560-025-09647-1 (PMC12102099; doi:10.1007/s12560-025-09647-1)
Supplement: Supplementary file 1 — Supplementary file1 (DOCX 35 KB) [file 12560_2025_9647_MOESM1_ESM.docx]

**Quantification and genotyping of norovirus in aerosols from wastewater treatment plants in Thailand**

Leera Kittigul^1*^, Kitwadee Rupprom^2^, Yuwanda Thongpanich^1^, Thanakrit Neamhom^3^, & Fuangfa Utrarachkij^1^

^1^Department of Microbiology, Faculty of Public Health, Mahidol University, Bangkok, Thailand. ^2^Department of Clinical Pathology, Faculty of Medicine Vajira Hospital, Navamindradhiraj University, Bangkok, Thailand.

^3^Department of Environmental Health Sciences, Faculty of Public Health, Mahidol University, Bangkok, Thailand.

^*^Corresponding author: Leera Kittigul

**Table S1.**  Specific primers and probes used in RT-qPCR and RT-nested PCR for detection of norovirus

| Norovirus  genogroup | Primer/  probe | Sequence (5’ 3’) | Polarity | Location | Reference |
| --- | --- | --- | --- | --- | --- |
| RT-qPCR | | | | | |
| GI | GITF | CGYTGGATGCGITTCCAT | + | 5291 – 5308 | Rupprom et al., 2018 |
|  | GITR | TCCTTAGACGCCATCATCATT | – | 5377 – 5357 |  |
|  | GIT-TP | FAM-TGGRCAGGAGAYCGC-MGB-BHQ | + | 5321 – 5335 |  |
| GII | QNIF2 | ATGTTCAGRTGGATGAGRTTCTCWGA | + | 5012 – 5037 | ISO, 2017 |
|  | COG2R | TCGACGCCATCTTCATTCACA | – | 5100 – 5080 |  |
|  | QNIFs | FAM-AGCACGTGGGAGGGCGATCG-TAMRA | + | 5042 – 5061 |  |
| RT-nested PCR | | | | | |
| GI | COG1F | CGYTGGATGCGNTTYCATGA | + | 5291 – 5310 | Kojima et al., 2002; Kageyama et al., 2003 |
|  | G1-SKR | CCAACCCARCCATTRTACA | – | 5671 – 5653 |  |
|  | G1-SKF | CTGCCCGAATTYGTAAATGA | + | 5342 – 5361 |  |
| GII | COG2F | CARGARBCNATGTTYAGRTGGATGAG | + | 5003 – 5028 |  |
|  | G2-SKR | CCRCCNGCATRHCCRTTRTACAT | – | 5389 – 5367 |  |
|  | G2-SKF | CNTGGGAGGGCGATCGCAA | + | 5046 – 5064 |  |

Mixed based in degenerate primers are as follows: Y=C or T; I= inosine; R= A or G; B = C, G or T; H = A, C or T; N = any base.

Nucleotide positions are taken from the reference norovirus GI (Norwalk virus 68; Genbank accession no. M87661) and norovirus GII (Camberwell virus; Genbank accession no. AF145896 or Lordsdale virus 93; Genbank accession no. X86557).

**References**

ISO (2017) INTERNATIONAL STANDARD 15216-1:2017—Microbiology of the food chain—Horizontal method for determination of hepatitis A virus and norovirus using real-time RT-PCR—Part-1: Method for quantification. 2017.

Kageyama, T., Kojima, S., Shinohara, M., Uchida, K., Fukushi, S., Hoshino, F. B.*,* et al. (2003). Broadly reactive and highly sensitive assay for Norwalk-like viruses based on real-time quantitative reverse transcription-PCR. *Journal of Clinical Microbiology*, *41*(4), 1548–1557.

Kojima, S., Kageyama, T., Fukushi, S., Hoshino, F. B., Shinohara, M., Uchida, K.*,* et al. (2002). Genogroup-specific PCR primers for detection of Norwalk-like viruses. *Journal of Virological Methods*, *100*(1–2), 107–114.

Rupprom, K., Chavalitshewinkoon-Petmitr, P., Diraphat, P., Vinje, J., & Kittigul, L. (2018). Development of one-step TaqMan quantitative RT-PCR assay for detection of norovirus genogroups I and II in oyster. *The Southeast Asian Journal of Tropical Medicine and Public Health,* *49*(6), 1017–1028.

**Table S2.**  Specific primers and probe used in RT-qPCR and RT-nested PCR for detection of rotavirus

| Primer/ probe | Sequence (5’ 3’) | Polarity | Location | Reference |
| --- | --- | --- | --- | --- |
| RT-qPCR | | | | |
| NSP3F | ACCATCTWCACRTRACCCTCTATGAG | + | 963 – 988 | Zeng et al., 2008 |
| NSP3R | GGTCACATAACGCCCCTATAGC | – | 1049 – 1028 |  |
| NSP3P | FAM-AGTTAAAAGCTAACACTGTCAAA-  MGB-BHQ | + | 995 – 1017 |  |
| RT-nested PCR | | | | |
| RV1 | GTCACATCAAATTCTAATCTAAG | – | 1061 –1036 | Gilgen et al., 1997 |
| RV2 | CTTTAAAAGAGAGAATTTCCGTCTG | + | 3 – 27 |  |
| RV3 | TGTATGGTATTGAATATACCAC | + | 50 – 71 |  |
| RV4 | ACTGATCCTGTTGGCCAWCC | – | 395 – 376 |  |

W=A or T.

Nucleotide positions are taken from the reference rotavirus (human group A rotavirus strain Wa, G1P[8]; Genbank accession no. KT694958).

**References**

Gilgen, M., Germann, D., Lüthy, J., & Hübner, P. (1997). Three-step isolation method for sensitive detection of enterovirus, rotavirus, hepatitis A virus, and small round structured viruses in water samples. *International Journal of Food Microbiology,* *37*(2-3), 189–199.

Zeng, S. Q., Halkosalo, A., Salminen, M., Szakal, E. D., Puustinen, L., & Vesikari, T. (2008). One-step quantitative RT-PCR for the detection of rotavirus in acute gastroenteritis. *Journal of Virological Methods,* *153*(2), 238–240.

**Table S3.** A total of 24 aerosol sample collection from 8 wastewater treatment plants (each 3 sites). Each sample was collected from each area for 5-mL collection vessel.

| No. | Collection date | Wastewater treatment plant (WWTP) | Sample code | Collection site |
| --- | --- | --- | --- | --- |
| 1 | 13 Sep 23 | A | AW01 | Entrance |
| 2 |  |  | AW02 | Treatment |
| 3 |  |  | AW03 | Exit |
| 4 | 25 Sep 23 | B | AW04 | Entrance |
| 5 |  |  | AW05 | Treatment |
| 6 |  |  | AW06 | Exit |
| 7 | 27 Sep 23 | C | AW07 | Entrance |
| 8 |  |  | AW08 | Treatment |
| 9 |  |  | AW09 | Exit |
| 10 | 29 Sep 23 | D | AW10 | Entrance |
| 11 |  |  | AW11 | Treatment |
| 12 |  |  | AW12 | Exit |
| 13 | 04 Sep 23 | E | AW13 | Entrance |
| 14 |  |  | AW14 | Treatment |
| 15 |  |  | AW15 | Exit |
| 16 | 06 Sep 23 | F | AW16 | Entrance |
| 17 |  |  | AW17 | Treatment |
| 18 |  |  | AW18 | Exit |
| 19 | 09 Oct 23 | G | AW19 | Entrance |
| 20 |  |  | AW20 | Treatment |
| 21 |  |  | AW21 | Exit |
| 22 | 16 Oct 23 | H | AW22 | Entrance |
| 23 |  |  | AW23 | Treatment |
| 24 |  |  | AW24 | Exit |

**Table S4.** Site of aerosol sample collection with norovirus-positive and rotavirus-negative samples using RT-qPCR and RT-nested PCR.

| No. | Sample code | Collection date | Collection site | Norovirus | Norovirus  Genotype | Rotavirus |
| --- | --- | --- | --- | --- | --- | --- |
| 1 | AW01 | 13 Sep 23 | Entrance | GI + GII | - | Negative |
| 2 | AW05 | 25 Sep 23 | Treatment | GII | - | Negative |
| 3 | AW08 | 27 Sep 23 | Treatment | GII | - | Negative |
| 4 | AW09 | 27 Sep 23 | Exit | GII | GII.21 | Negative |
| 5 | AW11 | 29 Sep 23 | Treatment | GII | GII.21 | Negative |
| 6 | AW12 | 29 Sep 23 | Exit | GII | - | Negative |
| 7 | AW14 | 04 Oct 23 | Treatment | GII | - | Negative |
| 8 | AW17 | 06 Oct 23 | Treatment | GI | - | Negative |
